# Treatment of Acromegaly: Are We Satisfied With the Current Outcome?

**DOI:** 10.1016/j.ebiom.2014.12.010

**Published:** 2014-12-20

**Authors:** Ferdinand Roelfsema

**Affiliations:** Department of Endocrinology and Metabolism, Leiden University Medical Center, Leiden, The Netherlands

Acromegaly is a rare progressive endocrine disorder, with characteristic symptoms, due to excessive growth hormone (GH) secretion from a pituitary adenoma. Even more rarely, the disease can be caused by hereditary syndromes (multiple endocrine neoplasia type 1, McCune–Albright syndrome, Carney complex, and familial acromegaly), ectopic tumors or caused by tumor-related excessive GHRH secretion. The reported incidence of acromegaly is 3–4 per one million per year and the prevalence is 60 to 70 per one million, without geographical and sex differences. The disease is likely under-diagnosed (Schneider et al., 2008). Acromegaly leads to multisystem damage, impaired quality of life, even in cured patients, and decreased life expectancy when not properly treated. Since it is reasonable to assume (although not proven) that both the duration of untreated disease and severity of GH excess contribute to the acromegaly-associated abnormalities and organ damage, efforts should be undertaken to detect patients in the population at an early stage, to improve diagnostic procedures and further to develop superior medicines and surgical techniques. Awareness of acromegaly by primary health care doctors as well as by non-endocrine specialists would likely shorten the delay in diagnosis, which is now roughly between 6 and 10 years. When the disease is suspected, the diagnosis is made on increased age-related serum insulin-like growth factor (IGF)-1 and insufficient suppression of serum GH during glucose loading.

Treatment options are surgery, medical treatment and radiation therapy. The current consensus is that surgery (presently mostly 2-D or 3-D endoscopic surgery, eventually with various forms of neuronavigation) should be performed as an initial treatment by an experienced surgeon in a center with expertise of acromegaly treatment. Normalization of GH and IGF-1 after the first surgical treatment of acromegaly is obtained in 61.2% (range 37–88), based on 32 studies (Roelfsema et al., 2011). Surgery-related pituitary insufficiency is low with 7% and the overall recurrence rate 4.9%, and not related to tumor size. A problem during surgery is the detection of small tumor remnants, especially tumor outgrowth in the cavernous sinus. Careful extended exploration is one of the strategies used. Other groups have introduced the intraoperative use of high resolution MRI with favorable results. Another approach, potentially improving direct results and late outcome in acromegaly, is the application of intraoperative imaging-guided surgery, targeted at the GHRH receptor in acromegaly, as presently in the investigational stage for detection carcinoma remnants during surgery (Chi et al., 2014).

Drugs for medical treatment of acromegaly are dopamine agonists, somatostatin analogs, GH-receptor-blocking agents, GH-receptor synthesis blocking agents, and GH-transcription blocking agents. At present long-acting forms of somatostatin analogs are widely used as GH-suppressive agents. The current clinically used slow-release analogs, octreotide and lanreotide, inhibit GH secretion via the somatostatin receptor subtypes 2 and 5. Although the most important effect of somatostatin analogs is the inhibition of tumor-derived GH and the subsequent fall in circulating liver-derived IGF-I, part of the peripheral effects are caused by the direct inhibition of IGF-I gene transcription via activation after binding to the somatostatin receptor. Multicenter studies have shown that disease activity is controlled in 40–60% of the patients (Roelfsema et al., 2005). Tumor volume reduction of GH adenoma with a weighted mean of 19.4% has been reported to occur in 62% of acromegalic patients during primary therapy with somatostatin analogs (Melmed et al., 2005). A recently marketed drug is pasireotide, which has binding affinities to all somatostatin receptor subtypes, except SST4. The long-acting form of pasireotide requires monthly injections, similar as the other registered long-acting somatostatin analogs. Several phase III clinical trials comparing the efficacy with other long-acting analogs are currently being performed (ClinicalTrials.gov NCT00600866, NCT00446082). All somatostatin analogs inhibit insulin secretion, but whether glucose intolerance or frank diabetes mellitus will be more frequent or severe with pasireotide than octreotide or lanreotide is not known yet. A potential very interesting drug (Octreolin®) uses the Transient Permeability Enhancer (TPE) technology. The TPE system causes temporary opening of the tight junctions of the small intestine epithelium, allowing the passage of octreotide (or any other drug) into the blood system. Currently, a multicenter trial is carried out (NCT01412424). If Octreolin is successful in this relative small competitive market, the treatment of persisting acromegaly is greatly simplified. The use of more effective GH- or IGF-I-suppressive drugs with this carrier system could further improve results.

The role of dopamine agonists is rather limited, and the drugs are mostly used as an adjunct to other forms of medical treatment if IGF-I normalization is not achieved. Cabergoline is the drug of choice and in its present dosage does not lead to cardiac valvular dysfunction. More effective is the GH-receptor blocking drug pegvisomant in normalizing IGF-I, especially when GH levels are not very high, as usually found in previously operated patients. Combining pegvisomant with long-acting somatostatin analogs is an effective way to improve results, permitting dose reduction of the expensive pegvisomant (van der Lely et al., 2012). Patients require careful monitoring of liver functions and the tumor remnant.

Several pharmaceutical companies have developed drugs aimed at blocking the IGF receptor, including GSK 1904529A and ATL 1103, an antisense drug. Although these drugs could be used potentially in acromegaly, they are primarily developed as adjunct drugs in cancer treatment (Tachas et al., 2006, Sabbatini et al., 2009).

A new concept in inhibiting GH secretion is the construction of targeted secretion inhibitors. These engineered proteins incorporate botulinum neurotoxin and a GHRH-receptor binding domain. After binding to the receptor, the complex is internalized in the endosome. Insertion of the translocation domain into the endosomal membrane allows the delivery of endopeptidase into the cell, leading to cleavage of the SNARE proteins (soluble N-ethylmaleimide-sensitive factor attachment protein receptor). SNARE proteins dock and fuse GH hormone-containing vesicles with the cell surface membrane, allowing subsequent GH secretion. Experimental data in rats have shown that this class of promising drugs is a potent inhibitor of GH secretion, but the present formulations are without GH-suppressive effect in human adenoma in vitro (Somm et al., 2012).

The place of radiation therapy in the treatment of acromegaly is rather limited, and currently is only used when all other options are unsuccessful in normalizing IGF-I. Radiation modalities include fractionated conventional external beam irradiation with a linear accelerator via a three-field or a rotational technique. The total dose is generally 40–50 Gy, and the fractional dose 1.8–2.0 Gy. The time interval required for normalization of GH depends on the initial level. The most frequent side effect is hypopituitarism in up to 50% of the patients after 10 years. The other treatment modality is radiosurgery. This technique is the precise stereotactic delivery of a single high radiation dose to a defined target with a steep dose gradient at the margin. Radiation may be given by the Gamma knife, a linear accelerator (Linac)-based system or proton beams (Minniti et al., 2011). Particularly, proton beam equipment is very expensive, but may be superior to other forms of radiation therapy. Nevertheless, long-term controlled studies comparing results of the different treatment forms are largely lacking.

In summary, there are several possible strategies for improving outcome of treatment of acromegaly. Even if patients are cured by expert surgery, many of them are still suffering from irreversible organ damage. In an ideal world the diagnosis should be made before gross and irreversible abnormalities occur. Awareness of this disease by primary care physicians may lead to earlier and more frequent diagnosis. Further improvement of endoscopic surgery tools with equipment for detection of small tumor remnants after removal of the gross tumor will improve the outcome of surgery. The second generation of somatostatin receptor blocking drugs and the GH-receptor blocking pegvisomant, although rather effective if GH levels are not high, should be replaced my more effective drugs. Several of these new classes of drugs with different action mechanisms are currently under development.
